# A novel nutritional immune risk score model for long-term prognosis in colorectal cancer using clustering and principal component analysis

**DOI:** 10.3389/fnut.2026.1734873

**Published:** 2026-04-15

**Authors:** Yanchun Shi, Yan Wang, Ting Sun, Lili Du, Yongqiang Lv, Ze Chen, Danshu Hao

**Affiliations:** 1 Department of Clinical Laboratory, Shanxi Province Cancer Hospital/Shanxi Hospital Affiliated to Cancer Hospital, Chinese Academy of Medical Sciences/Cancer Hospital Affiliated to Shanxi Medical University, Taiyuan, Shanxi, China; 2 Operations Management Department, Shanxi Province Cancer Hospital/Shanxi Hospital Affiliated to Cancer Hospital, Chinese Academy of Medical Sciences/Cancer Hospital Affiliated to Shanxi Medical University, Taiyuan, Shanxi, China; 3 Central Laboratory, Shanxi Province Cancer Hospital/Shanxi Hospital Affiliated to Cancer Hospital, Chinese Academy of Medical Sciences/Cancer Hospital Affiliated to Shanxi Medical University, Taiyuan, Shanxi, China; 4 Department of Clinical Nutrition, First Hospital of Shanxi Medical University/First Clinical Medical College of Shanxi Medical University, Taiyuan, China

**Keywords:** colorectal cancer, maximum tumor diameter, nutritional immune risk score model, PNI, prognosis, tumor markers, unsupervised learning

## Abstract

**Background:**

Survival outcomes among patients with colorectal cancer (CRC) often differ despite identical disease stages, partly due to variations in nutritional and immune status. Malnutrition can impair immune defense, exacerbate inflammatory responses, and influence tumor progression, ultimately contributing to a poorer prognosis. However, current clinical prognostic systems rarely integrate nutritional immune indicators with tumor biomarkers, limiting the application of nutritional intervention in CRC management. This study aimed to develop a nutritional immune risk score (NIRS) model to improve long-term prognostic evaluation in patients with CRC.

**Methods:**

In this retrospective study, 892 inpatients with primary CRC who underwent curative resection in 2017 were included and followed until 2023. Unsupervised learning was applied to nutritional and tumor biomarkers for feature extraction and patient stratification. K-means clustering was used to identify subgroups, and principal component analysis was used to derive composite features, which were then used to construct the NIRS model for long-term prognostic assessment.

**Results:**

Four variables—prognostic nutritional index (PNI), carcinoembryonic antigen (CEA), carbohydrate antigen 19–9 (CA19-9), and carbohydrate antigen 72–4 (CA72-4)—were selected for model construction. The final model was defined as: NIRS = 0.572 × PNI – 0.101 × CEA – 0.412 × CA19-9 – 0.028 × CA72-4. Using an optimal cutoff value of 21.34, patients were stratified into a low-risk group and a high-risk group. The Kaplan–Meier analysis showed that patients in the low-risk group had significantly better overall survival than those in the high-risk group (*p* < 0.001). Multivariable Cox regression analysis indicated that the high-risk group had a 1.72-fold higher mortality risk than the low-risk group (HR = 1.72, 95% CI: 1.34–2.21, *p* < 0.001). In addition, PNI was negatively correlated with maximum tumor diameter in both survivors and non-survivors (survivors: *r* = −0.434, *p* < 0.001; non-survivors: *r* = −0.214, *p* < 0.001). Locally estimated scatterplot smoothing (LOESS) analysis further demonstrated that among patients with PNI ≥ 50, survivors had smaller tumors than non-survivors, whereas the opposite pattern was observed among patients with PNI < 50.

**Conclusion:**

We developed a novel NIRS for long-term prognostic assessment in patients with CRC. The NIRS model demonstrated robust risk stratification and potential clinical utility. PNI may serve as a complementary factor to refine risk classification, and its interaction with maximum tumor diameter may improve the sensitivity and precision of prognostic assessment across different nutritional immune states.

## 1 Introduction

The incidence of colorectal cancer (CRC) ranks third worldwide and second in China (1, 2). Curative resection is a common surgical procedure for treating patients with CRC, and accurate prognostic assessment is essential for improving postoperative survival outcomes. Currently, prognostic assessment mainly relies on tumor-related indicators, such as tumor–node–metastasis stage (TNM stage, where T indicates primary tumor size, N indicates regional lymph node involvement, and M indicates distant metastasis). However, increasing evidence indicates that patients with identical disease stages can exhibit considerable heterogeneity in survival outcomes (3). Notably, patients with CRC at stage IIB or IIIC may have worse survival than those with stage IIIA (4, 5). This “stage paradox” underscores the importance of considering factors beyond the overall tumor stage when assessing patient prognosis. These factors include nutritional status, immune function, tumor-related characteristics, and genetic markers. To maximize the capture of associations among these factors, researchers have increasingly applied unsupervised learning approaches. Such methods can reveal biologically significant patterns within complex data without prior assumptions and provide an important complement to existing prognostic systems.

Malnutrition and impaired immune function are strongly associated with poorer prognosis in patients with CRC (6, 7). The prognostic nutritional index (PNI) is a simple blood-based score used to assess a patient’s nutritional and immune status, reflecting their overall health and predicting the risk of complications or mortality. Lower PNI values indicate malnutrition and immunosuppression, which are closely correlated with increased postoperative complications, a higher risk of tumor recurrence, and reduced survival rates (8–10). Multiple studies have confirmed that PNI is an independent prognostic factor in CRC (11, 12). Furthermore, serum tumor markers play an indispensable role in the prognostic management of tumors. Carcinoembryonic antigen (CEA), carbohydrate antigen 19–9 (CA19-9), and carbohydrate antigen 72–4 (CA72-4) are the three most widely used serum markers, reflecting tumor burden, metastasis risk, and invasiveness in digestive tract adenocarcinoma (13). Recent evidence has suggested that elevated levels of these markers are associated with alterations in the tumor immune microenvironment, metabolic dysfunction, systemic inflammation, and overall prognosis, indicating their broader biological significance beyond conventional tumor monitoring (13–15). Although the PNI and serum tumor markers show distinct aspects of host condition and tumor characteristics, respectively, integrated quantitative models that combine nutritional immune indices with serum marker profiles remain limited. Therefore, developing a novel nutritional immune model may enhance the accuracy of prognostic assessment in patients with CRC and provide additional insights into the interaction between host status and tumor behavior.

Clustering algorithms and principal component analysis (PCA), as unsupervised learning methods, are widely used for tumor subtype identification and risk stratification. Clustering algorithms can categorize potential biological subtypes based on feature similarity, revealing heterogeneity among patients (16). PCA, as a dimensionality-reduction method, extracts the most representative directions of variation in the features, thereby simplifying the data structure and enhancing the stability of subsequent modeling (17). In recent years, the application of clustering algorithms and PCA has advanced, with standardized protocols now available to address multiple technical challenges, such as selecting input features, determining the optimal number of clusters, and validating subtype stability. Several studies have attempted to integrate clustering algorithms with PCA to construct models and extract key features (18–20). However, determining the optimal strategy for combining these two methods and analyzing the relationships between the identified subtypes and clinical outcomes remain key challenges in model construction.

To construct a novel model for prognostic assessment of patients with CRC based on nutritional immunity, we established a comprehensive dataset including preoperative nutritional and immune indicators, clinical information, tumor characteristics, and follow-up data from 892 patients undergoing curative resection. We applied an unsupervised learning approach to nutritional and tumor biomarkers for feature extraction and patient stratification. K-means clustering was used to group patients into subtypes based on similarities in their nutritional and tumor biomarker profiles. PCA was subsequently applied to derive quantitative composite features from the clustering results, providing interpretable numerical representations of each subtype while preserving key variations among patients. This approach allows complex, multidimensional biomarker data to be simplified for downstream modeling, facilitates comparison across subtypes, and provides a standardized way to capture patient heterogeneity. These features were integrated to construct a nutritional immune risk score (NIRS) model, enabling objective prognostic assessment and stratification of risk levels for CRC patients.

## 2 Methods

### 2.1 Study population

A total of 892 inpatients with primary CRC who underwent curative resection between January and December 2017 were included in this retrospective study. The study population was of Chinese descent and met the following criteria: age ≥ 18 years, primary CRC confirmed by pathological examination, and availability of complete clinicopathological and follow-up data. Patients were excluded if they met the following criteria: receipt of neoadjuvant radiotherapy or chemoradiotherapy before surgery, history of relevant colorectal surgery prior to study enrollment, past or concurrent history of other malignancies, discontinuation of treatment after diagnosis, recurrence after surgery, incomplete clinical data, or loss to follow-up (Supplementary Flowchart S1).

### 2.2 Data collection and follow-up

Preoperative clinical data were retrieved from the hospital’s electronic medical record system, including patient-related variables, tumor characteristics, and laboratory parameters. Patient-related variables included age, sex, height, weight, smoking status, alcohol consumption status, and medical history of hypertension, diabetes, and other comorbidities. Tumor characteristics included TNM stage (based on the 8th edition of the American Joint Committee on Cancer [AJCC] staging system), tumor location (colon or rectum), presence of perineural invasion or vascular cancer thrombus, and maximum tumor diameter. Laboratory parameters were obtained from blood samples taken within 1 week before the surgery and included serum albumin, lymphocyte, CEA, CA19-9, and CA72-4. The PNI was calculated as follows:


$$\begin{array}{c} \mathrm{PNI}=\text{serum albumin level}\;(g/L)+\\ 5\times \text{peripheral blood lymphocyte count}\;(10^{9}/L) \end{array}$$

The final date of follow-up was 31 December 2023. The time origin was the date of surgery. Patients alive at the last follow-up were censored at the date of the last contact. Follow-up was conducted through a combination of telephone interviews, outpatient visits, and routine inpatient reviews to ensure data completeness and accuracy. Follow-up data included vital status, tumor recurrence or distant metastasis, and details of subsequent treatment strategies, enabling comprehensive assessment of disease progression and clinical outcomes.

### 2.3 Data processing and modeling

**Figure 1:**
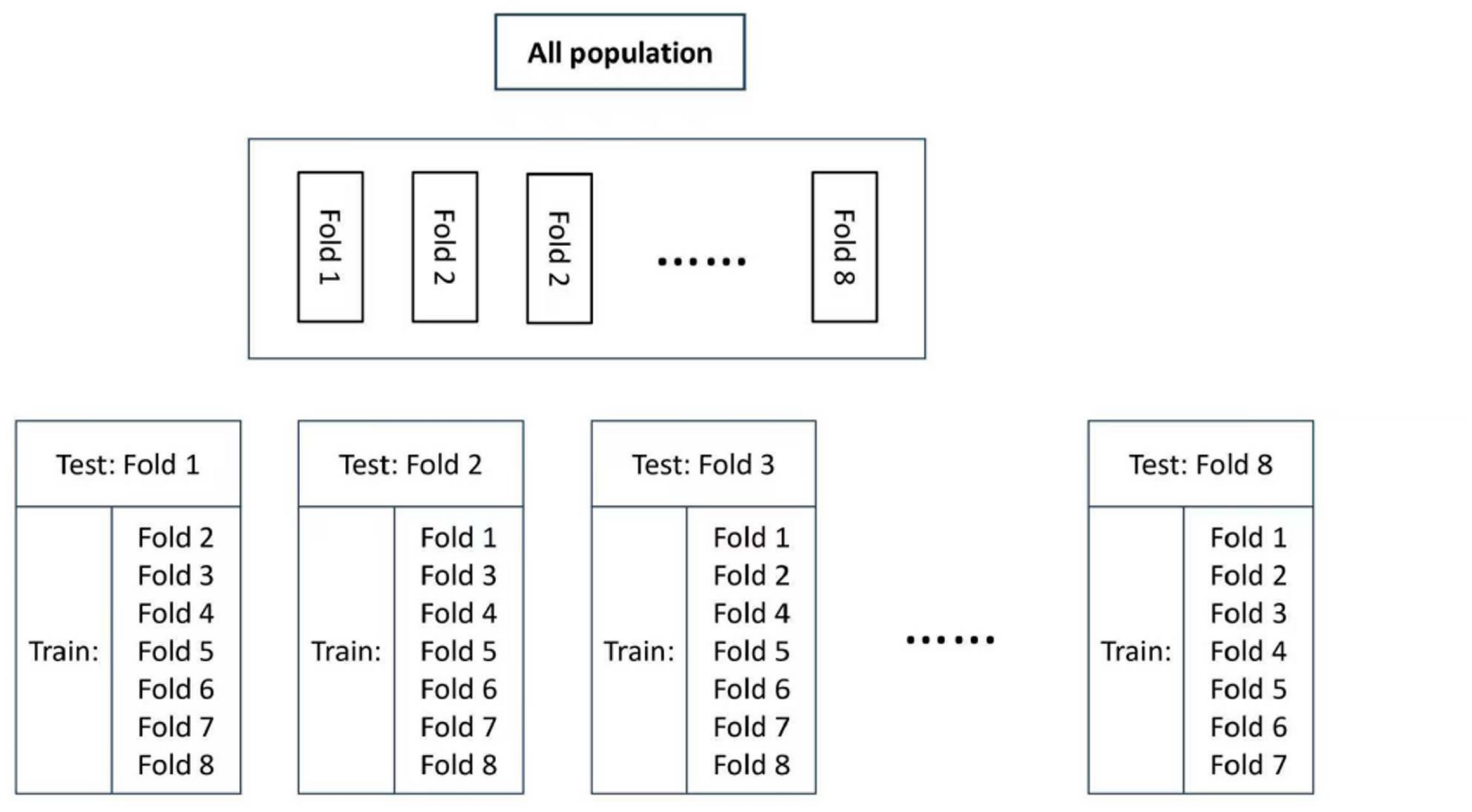
Eight-fold cross-validation for training and validating predictive models.

Winsorization was used to manage outliers in the raw dataset. For each variable, we calculated the first (Q1) and third (Q3) quartiles and the interquartile range (IQR = Q3 − Q1). Values below Q1–1.5 × IQR were set as the lower bound, and values above Q3 + 1.5 × IQR were set as the upper bound, obtaining the processed dataset. The Wilcoxon test was used to assess the consistency of distributions between the raw and processed data. Then, the processed data were standardized using Z-score and randomly divided into eight folds. In each iteration, one fold served as the test set, while the remaining seven folds formed the training set for cross-validation (Figure 1). During model training, the K-means clustering algorithm combined with PCA was applied to each training set to extract key component scores. The optimal cutoff point of component scores was determined using the Youden index. Receiver operating characteristic (ROC) analysis was used to evaluate the consistency of results between K-means clustering and PCA. For each test set, the cutoff value and model established from the training set were further evaluated using the Kolmogorov–Smirnov (K-S) test and the chi-squared test to verify the generalizability of the model.

Finally, the selected features were integrated for clustering and modeling using the elbow method and K-means clustering on the full processed dataset. Cluster quality was assessed using the silhouette coefficient. Visualization was performed using Uniform Manifold Approximation and Projection (UMAP) dimensionality reduction to examine distribution patterns and confirm cluster boundaries. PCA was subsequently used to further quantify clustering patterns and construct the final model. In addition, the relationship between the PNI and the maximum tumor diameter was explored using a linear regression analysis. To reduce noise, locally estimated scatterplot smoothing (LOESS; *n* = 30) was applied by sequentially shifting the smoothing window across the continuous PNI range. The mean maximum tumor diameter within each PNI interval was calculated for both groups, and the difference between the groups was used for dynamic comparison.

### 2.4 Statistical analysis

Statistical analysis was performed using R software (version 4.4.1). Data processing and analysis were performed using the R packages, including dplyr, tidyr, reshape2, survival, survminer, and car.

The normality of continuous variables was assessed using the Shapiro–Wilk test. Normally distributed variables were expressed as mean ± standard deviation (SD), and intergroup differences were compared using the independent samples *t*-test. Non-normally distributed variables were expressed as median and interquartile range (median [IQR]), and intergroup comparisons were performed using the Wilcoxon rank-sum test. Categorical variables were expressed as frequency (%), and intergroup differences were assessed using the chi-squared test or Fisher’s exact test, as appropriate. Pearson’s or Spearman’s correlation analysis was used to evaluate associations between continuous variables. The K-S test was used to compare distributions between the groups. ROC curve analysis was used to assess the discriminatory ability of variables or models, and the optimal cutoff value was determined using the Youden index.

Survival analysis was performed using the Kaplan–Meier method, and survival differences were assessed using the log-rank test. A Cox proportional hazards regression model was constructed to identify independent prognostic factors, with results reported as hazard ratios (HRs) and 95% confidence intervals (CIs). The concordance index (C-index) was calculated to assess the model’s predictive accuracy, and multicollinearity was assessed using the variance inflation factor (VIF). All figures were generated using the R packages, including ggplot2, cowplot, pheatmap, and pROC. A two-sided *p*-value of < 0.05 was considered statistically significant.

## 3 Results

### 3.1 Population characteristics

A total of 892 patients with CRC were included in this retrospective cohort study. Table 1 summarizes the characteristics of the study population. Among the patients, 53.6% had rectal cancer and 46.4% had colon cancer. The mean age was 60.83 ± 11.48 years, and males accounted for 57.2% of the cohort. The mean body mass index (BMI) was 23.64 ± 3.32 kg/m^2^. The mean maximum tumor diameter was 4.69 ± 1.81 cm. The distribution of TNM stages I–IV was 17.5%, 36.9%, 39.1%, and 6.5%, respectively. Regarding pathological features, vascular cancer thrombus and perineural invasion were observed in 15.2% and 7.5% of patients, respectively. We further described the nutritional immune indicators and tumor markers of the patients. The PNI showed a normal distribution, whereas CEA, CA19-9, and CA72-4 exhibited right-skewed distributions. The mean PNI value was 50.94 ± 6.08. The median (IQR) values of CEA, CA19-9, and CA72-4 were 2.17 (0.86–5.66), 13.55 (7.66–25.40), and 2.27 (0.94–5.59), respectively. At the end of the 6-year postoperative follow-up, 268 patients (30.0%) had died, whereas 624 patients (70.0%) were alive.

**Table 1:** Characteristics of the study population.

Indicators	All (*n* = 892)
Age (years)	60.83 ± 11.48
Age<65, *n* (%)	551 (61.8%)
Age≥65, *n* (%)	341 (38.2%)
Sex, *n* (%)	
Male	510 (57.2%)
Female	382 (42.8%)
BMI, mean ± SD (kg/m^2^)	23.64 ± 3.32
Primary site of cancer, *n* (%)	
Rectum	478 (53.6%)
Colon	414 (46.4%)
Survival status, *n* (%)	
Death	268 (30.0%)
Alive	624 (70.0%)
Maximum tumor diameter (cm)	4.69 ± 1.81
TNM, *n* (%)	
I	156 (17.5%)
II	329 (36.9%)
III	349 (39.1%)
IV	58 (6.5%)
Vascular cancer thrombus, *n* (%)	
Yes	136 (15.2%)
No	756 (84.8%)
Perineural invasion, *n* (%)	
Yes	67 (7.5%)
No	825 (92.5%)
Bowel obstruction, *n* (%)	
Yes	187 (21.0%)
No	705 (79.0%)
Indicators for NIRS Model	
PNI, mean ± SD	50.94 ± 6.08
CEA, median (IQR)	2.17 (0.86–5.66)
CA19-9, median (IQR)	13.55 (7.66–25.40)
CA72-4, median (IQR)	2.27 (0.94–5.59)

### 3.2 Feature extraction and clustering of CEA, CA19-9, CA72-4, and PNI using K-means and PCA based on cross-validation

**Figure 2:**
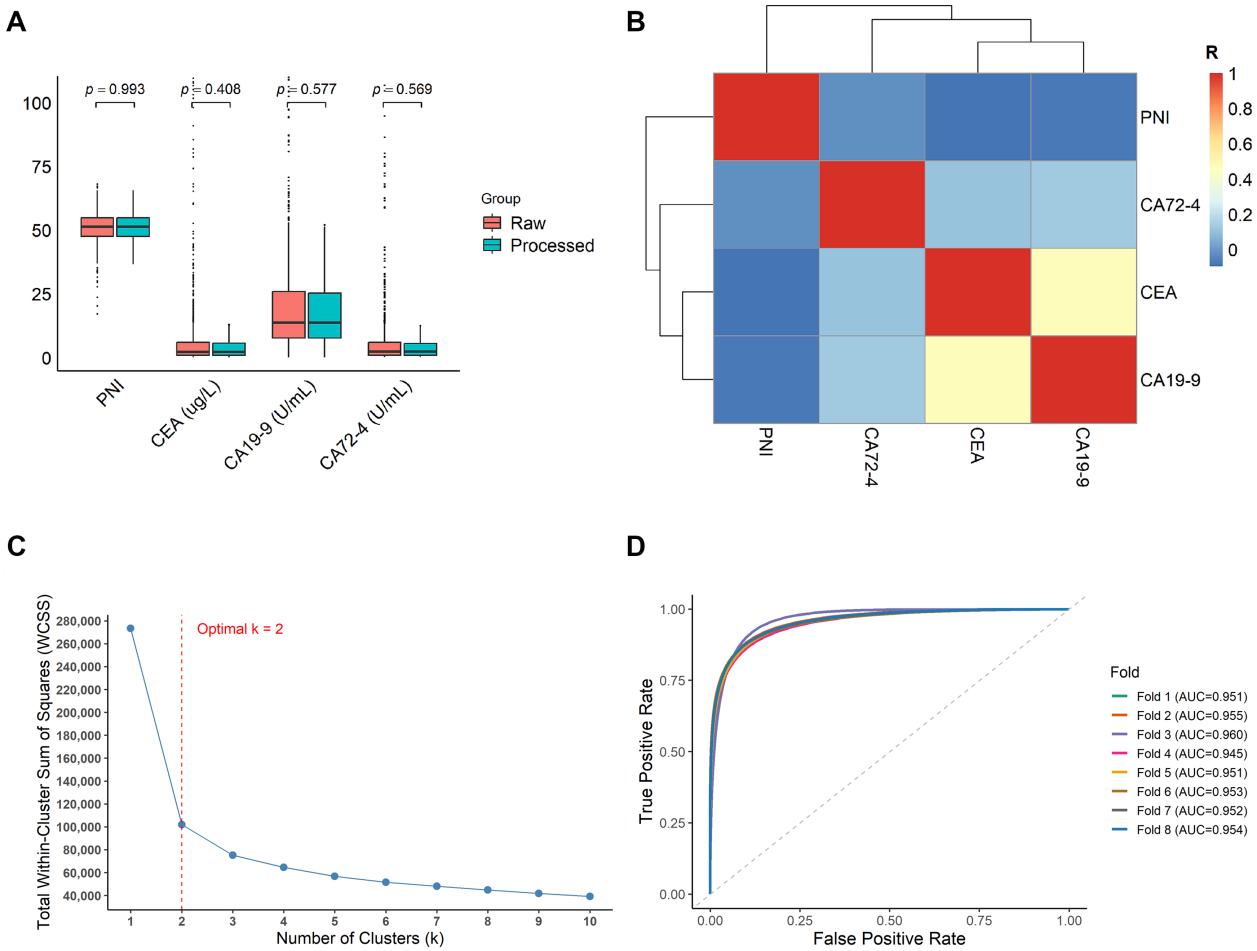
Data processed and unsupervised training before developing the model. **(A)** The Wilcoxon test shows that the data of PNI, CEA, CA19-9, and CA72-4 are not significantly different between the raw data and the processed data. **(B)** The matrix diagram shows the correlation among the four features. Pearson’s correlation coefficient (*R*) becomes higher and higher as the color changes from dark blue to dark red. An *R* value between 0.4 and 0.7 indicates a moderate correlation. **(C)** The elbow method shows the number of clusters. The red dashed line shows that the optimal number of clusters is two (*k* = 2). **(D)** The ROC analysis shows excellent classification performance in 8 folds, respectively. An AUC value of above 0.9 represents outstanding classification.

After feature extraction in the processed dataset, CEA, CA19-9, CA72-4, and PNI were finally determined as core indicators to define clusters. Following the Wilcoxon test, none of the *p*-values for CEA, CA19-9, CA72-4, and PNI between the raw and processed data were statistically significant, suggesting that the processed data was stable and consistent with the raw data (Figure 2A). The correlation matrix further showed that, except for a moderate positive correlation between CEA and CA19-9, correlations among the remaining variables were weak, suggesting a relatively high degree of independence among the variables (Figure 2B). This favorable data structure supported subsequent clustering analysis and PCA.

Then, the elbow method was used to determine the optimal number of clusters in the processed dataset. Based on the within-cluster sum of squares (WCSS) across different values of k, the elbow was identified at *k* = 2, which was therefore selected as the optimal number of clusters for this study (Figure 2C). The processed data were then randomly divided into eight folds, with each fold serving as the test set once, while the remaining seven folds served as the training set for cross-validation. After running the algorithm, the ROC for K-means clustering and PCA in the training sets showed that all AUC values exceeded 0.95, with a mean AUC of 0.953 across the eight folds, indicating high consistency between the two methods and excellent classification performance (Figure 2D). Finally, the trained model was evaluated in the test sets. The K-S test and chi-squared test showed that nearly all *p*-values across the folds were >0.05, suggesting that distributions of continuous scores and categorical variables between the training and test sets were not significantly different (Supplementary Table S1). These results indicate that the model demonstrated strong stability and generalizability.

### 3.3 NIRS model shows nutritional immune status and tumor burden in CRC patients

**Figure 3:**
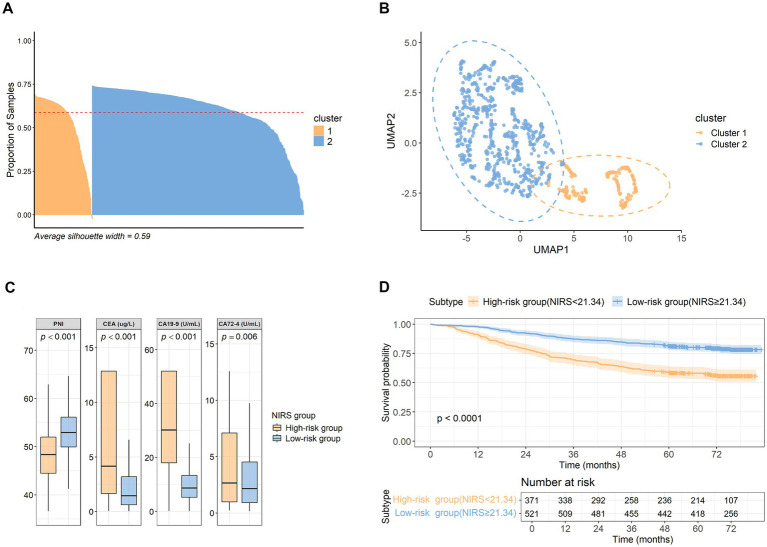
Clustering algorithm and PCA for developing the NIRS model. **(A)** Cluster 1 (orange) consists of 192 patients, and cluster 2 (blue) consists of 700 patients. The red dashed line represents the value of the silhouette coefficient. A silhouette coefficient of above 0.5 represents reasonable clustering. **(B)** The UMAP shows the boundary of clusters. The orange dots represent cases in cluster 1, and the blue dots represent cases in cluster 2. Nearly no overlap between the two clusters indicates the clarity of the identified groups. **(C)** PNI, CEA, CA19-9, and CA72-4 are significantly different between the high-risk group (*n* = 371, NIRS <21.34) and the low-risk group (*n* = 521, NIRS ≥21.34) according to the NIRS model. The box plots show the median (horizontal line) and the 25th and 75th percentiles. *p*-values are provided at the top of the box plot for each feature. **(D)** The Kaplan–Meier survival curve shows that the low-risk group has better survival outcomes. The *p*-value is provided at the bottom of the graph.

Based on *k* = 2, the dataset consisting of CEA, CA19-9, CA72-4, and PNI was divided into two clusters: cluster 1 (*n* = 192) and cluster 2 (*n* = 700). The silhouette coefficient was 0.59, indicating a reasonably well-defined cluster structure with good consistency within clusters (Figure 3A). UMAP visualization further illustrated the proximity of data points within clusters and the overall separation between the two clusters (Figure 3B). PCA was used to extract the principal component scores. To balance the contribution of each feature to the model, PC1 and PC2 were combined to construct the nutritional immune risk score (NIRS) model. The loading coefficients of the variables on PC1 were as follows: PNI: 0.032, CEA: –0.136, CA19-9: −0.990, and CA72-4: −0.036 (Supplementary Table S2). The loading coefficients of the variables on PC2 were as follows: PNI: 0.996, CEA: –0.073, CA19-9: 0.043, and CA72-4: −0.021 (Supplementary Table S2). A weighted combination of PC1 and PC2 was used to construct the NIRS model, with weights of 0.44 and 0.56, respectively. The final model was defined as follows:


$$\begin{array}{l} \text{NIRS}=0.572\times \mathrm{PNI}–0.101\times \mathrm{CEA}–0.412\times \mathrm{CA}19-9\\ –0.028\times \mathrm{CA}72-4 \end{array}$$

ROC analysis identified the optimal cutoff value as 21.34. Based on this threshold, patients were classified into a low-risk group (NIRS ≥21.34) and a high-risk group (NIRS <21.34). The high-risk group showed significantly lower PNI levels and higher levels of CEA, CA19-9, and CA72-4 (Figure 3C, PNI: *p* < 0.001; CEA: *p* < 0.001; CA19-9: *p* < 0.001; CA72-4: *p* = 0.006). Overall, the high-risk group tended to exhibit higher levels of tumor burden and poor nutritional immune status, whereas the low-risk group showed the opposite pattern.

### 3.4 NIRS model has independent prognostic value for long-term survival in CRC patients

The Kaplan–Meier survival analysis showed that the low-risk group’s survival probability declined more slowly over time than that of the high-risk group, and its long-term survival performance was better (Figure 3D, *p* < 0.001). Patients with CRC in the low-risk group, characterized by better nutritional immune status and lower tumor burden, tended to have higher overall survival.

**Figure 4:**
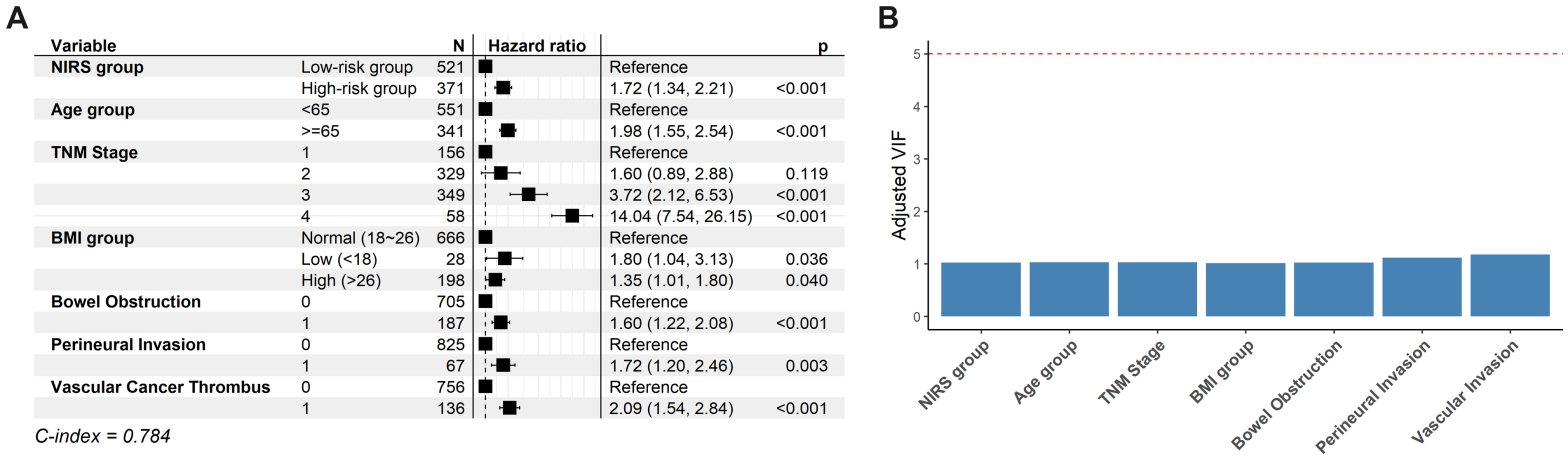
NIRS model has independent long-term prognostic value for survival in CRC patients. **(A)** The forest plot summarizes the adverse factors for CRC. NIRS is an independent adverse factor for CRC. The leftmost column shows the name of variables, the middle column shows the hazard ratio (HR) for each of these variables, and the rightmost column shows the value of HR, confidence intervals, and *p*-value. A C-index closer to 1 indicates better model performance. **(B)** The variance inflation factor (VIF) shows multicollinearity on each variable. A VIF value closer to 1 indicates nearly no correlation.

To further evaluate the prognostic value of the NIRS model, a Cox proportional hazards model was constructed with adjustment for multiple covariates, including age group, TNM stage, BMI, bowel obstruction, perineural invasion, and vascular cancer thrombus. The results showed that, after adjusting for these variables, the mortality risk for patients with CRC in the high-risk group was 1.72 times higher than that for patients in the low-risk group (Figure 4A, HR = 1.72, 95% CI: 1.34–2.21, *p* < 0.001). BMI was identified as a risk factor when it was lower than 18 or higher than 26 (HR = 1.8, *p* = 0.036; HR = 1.35, *p* = 0.04). In addition, age ≥65 years (HR = 1.98, *p* < 0.001), TNM stage III (HR = 3.72, *p* < 0.001), TNM stage IV (HR = 14.04, *p* < 0.001), bowel obstruction (HR = 1.6, *p* < 0.001), perineural invasion (HR = 1.72, *p* = 0.003), and vascular cancer thrombus (HR = 2.09, *p* < 0.001) were also identified as independent adverse prognostic factors (Figure 4A). The C-index was 0.784, indicating good prognostic performance of the model (Figure 4A). Collinearity among variables in the Cox model was assessed with the VIF. The VIF values were: NIRS group: 1.023, age group: 1.029, TNM stage: 1.029, BMI: 1.015, bowel obstruction: 1.012, perineural invasion: 1.110, and vascular cancer thrombus: 1.166 (Figure 4B). All VIF values were close to 1, indicating no significant multicollinearity among the variables and confirming the stability and reliability of the model. Overall, the NIRS model showed independent prognostic value for long-term survival in patients with CRC.

### 3.5 PNI and maximum tumor diameter correlate according to survival status

**Figure 5:**
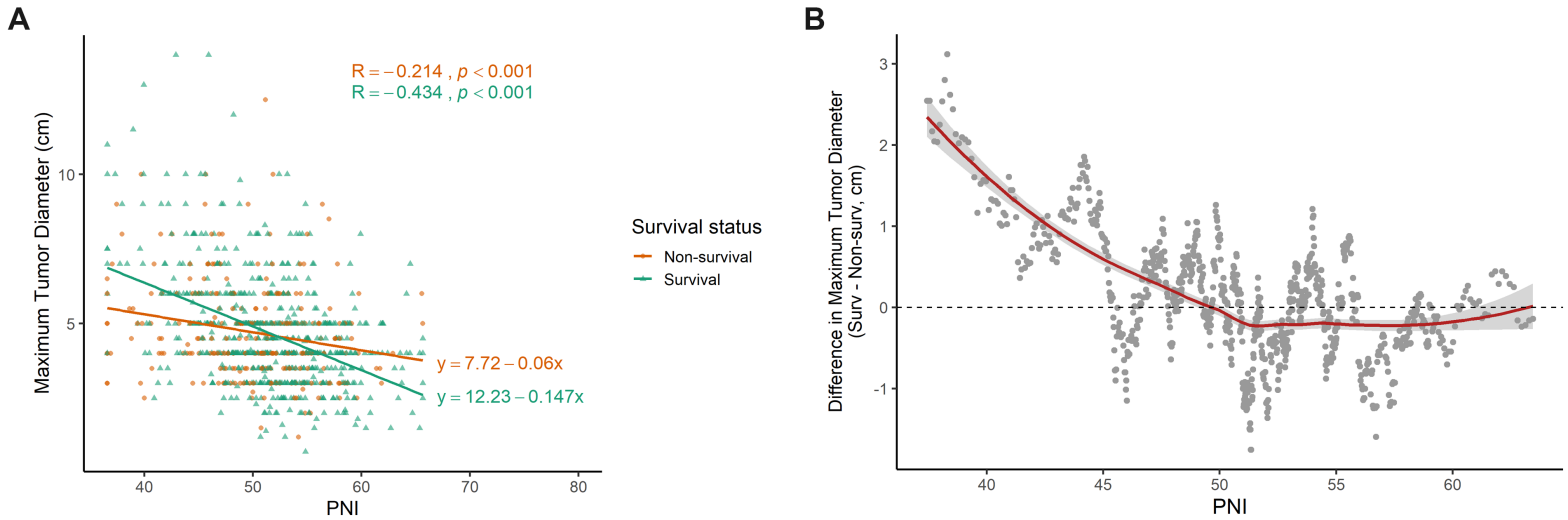
PNI and maximum tumor diameter correlation in CRC patients. **(A)** The dot plots show the correlations between the PNI and maximum tumor diameter in the survival group (*n* = 624, green dots) and the non-survival group (*n* = 268, orange dots). Pearson’s correlation coefficient (R) and the significance (*p*) are provided at the top of the graph. Variables and their units are indicated on the corresponding axes. **(B)** The fitting curve was plotted with PNI as the horizontal axis and the differences in the maximum tumor diameter (survivors – non-survivors) as the vertical axis. The gray dots represent the different points calculated within the sliding interval with a window of 30 cases, sliding the window across the data. The dots above 0 represent the bigger maximum tumor diameter in survivors. The dots below 0 represent the bigger maximum tumor diameter in non-survivors.

Based on the final survival outcomes after the 6-year postoperative follow-up, patients were divided into a survival group and a non-survival group. Negative correlations between the PNI and the maximum tumor diameter were observed in both groups, indicating that this relationship was present across all patients (Figure 5A, non-survival: *r* = −0.214, *p* < 0.001; survival: *r* = −0.434, *p* < 0.001). However, the maximum tumor diameter decreased faster with increasing PNI in the survival group than in the non-survival group (Figure 5A, survival: y = 12.23–0.147x; non-survival: y = 7.72–0.06x, *p* < 0.001). For each 1-unit increase in the PNI, the maximum tumor diameter decreased by approximately 0.087 cm more in the survival group than in the non-survival group, suggesting that survivors were more responsive to changes in the PNI.

To further explore this relationship, LOESS analysis was used to dynamically compare the difference in maximum tumor diameter between the survival and non-survival groups. The results showed that as PNI increased, the difference in tumor size decreased rapidly and gradually stabilized. The fitted curve (red curve) and its 95% CI (gray zone) indicated that this trend was statistically robust (Figure 5B). Interestingly, when the value of PNI was ≥50, the difference approached zero and then became a stable negative value. In other words, with higher nutritional immune status, survivors had smaller tumor diameters than non-survivors. However, when the value of PNI was <50, the difference became positive, indicating that under lower nutritional immune status, tumors in the survival group were larger than those in the non-survival group. To exclude the confounding effect of stage mix, we further performed a stratified analysis. When the value of PNI was <50, the results consistently supported that survivors exhibited a larger maximum tumor diameter than non-survivors across stages I–IV (Supplementary Table S3, stage I: *p* = 0.737; stage II: *p* = 0.758; stage III: *p* = 0.001; stage IV: *p* = 0.819). This finding suggests a complex relationship between nutritional immune status and tumor burden in patients with CRC.

## 4 Discussion

Substantial evidence has shown that immune function and nutritional status play key roles in tumor progression and prognosis in patients with CRC. Researchers have attempted to identify reliable indicators that comprehensively assess the relationship between nutritional immune status and tumor burden to guide clinical treatment strategies and evaluate the prognosis of CRC patients. However, there is still no consensus protocol. In our study, we identified PNI, CEA, CA19-9, and CA72-4 as significant contributing factors and integrated them into a novel NIRS model for patients with CRC. Unlike traditional single-indicator accumulation, our model extracts the maximum information through dimensionality reduction while retaining intrinsic correlations among variables, thereby enhancing the efficiency of information integration and the stability of predictions. The NIRS model effectively quantified risk in patients with CRC, with those classified in the low-risk group showing significantly better long-term survival outcomes. Therefore, the NIRS model may serve as a potential tool for preoperative risk stratification and postoperative survival prediction. Interestingly, we observed a negative correlation between the PNI and the maximum tumor diameter in patients with CRC. Among patients with good nutritional immune status, survivors tended to have smaller maximum tumor diameters compared to non-survivors. However, among those with poor nutritional immune status, survivors exhibited larger tumor sizes than non-survivors. This suggests that for those with a poor nutritional immune status, a larger tumor size does not necessarily correspond to a worse prognosis.

The design of the NIRS model underscores the explanatory power of nutritional immune feature extraction and the rationale of the modeling strategy. The PNI exhibits both the nutritional status and immune reserve in patients (12). CEA and CA19-9, which are commonly used as tumor markers for digestive tract adenocarcinoma, exhibit dynamic changes in tumor biological activity (13). CA72-4, as an additional marker, shows relative specificity for gastrointestinal tumors (21). Together, these features capture core information regarding systemic nutritional immune status and the biological behavior of tumors in patients with CRC. Before model construction, the Wilcoxon test and the correlation matrix analysis confirmed that the processed data maintained stability relative to raw data and exhibited an appropriate structure for subsequent clustering analysis and model training (Figures 2A,B). Using eight-fold cross-validation, the NIRS model was trained on the training sets, and all AUC values exceeded 0.95, indicating excellent model classification performance (Figure 2D). Furthermore, the K-S and chi-squared tests showed that nearly all *p*-values across folds were >0.05, indicating strong model stability and generalizability (Supplementary Table S1). The NIRS model was constructed as a weighted linear combination of the first two principal components, thereby integrating multi-dimensional biological information while preserving the differential contributions of each feature (Supplementary Table S2). These results support the applicability of the NIRS model to clinical data.

The final NIRS formula was defined as follows: NIRS = 0.572 × PNI – 0.101 × CEA – 0.412 × CA19-9 – 0.028 × CA72-4. By integrating these four features, the NIRS model simultaneously captures two critical biological signals: host nutritional immune status and tumor behavior. A lower NIRS value indicates a higher risk of an elevated tumor burden and impaired nutritional immune status. In clinical practice, patients with CRC do not always present with clearly identifiable risk profiles; for example, they may not simultaneously exhibit lowered PNI and elevated CEA, CA19-9, and CA72-4 beyond medical reference ranges. Therefore, the NIRS model provides a valuable tool for quantifying risk in patients with heterogeneous clinical characteristics. Further analysis identified 21.34 as the optimal cutoff value for risk stratification. Patients in the high-risk group showed significantly higher tumor marker levels and poorer nutritional status (Figure 3C). Conversely, patients in the low-risk group tended to have lower tumor marker levels and better nutritional immune status (Figure 3C). Statistical comparisons of PNI, CEA, CA19-9, and CA72-4 between the two groups further confirmed these differences (Figure 3C, PNI: *p* < 0.001, CEA: *p* < 0.001, CA19-9: *p* < 0.001, CA72-4: *p* = 0.006).

Survival analysis showed that the low-risk group had significantly higher overall survival than the high-risk group (Figure 3D, *p* < 0.001). Notably, the NIRS model presented robust long-term prognostic performance. The Cox regression analysis further confirmed that the high-risk group had a significantly higher mortality risk than the low-risk group (Figure 4A, HR = 1.72, 95% CI: 1.34–2.21, *p* < 0.001), suggesting that the NIRS model serves as an independent predictor of overall survival in patients with CRC. In addition, age ≥65 years, TNM stages III–IV, BMI ≥ 26 or <18 kg/m^2^, bowel obstruction, perineural invasion, and vascular cancer thrombus were identified as independent adverse prognostic factors. These findings are consistent with previous large-scale CRC cohort studies, thereby reinforcing the biological plausibility of the model results (22–24). Notably, in our Cox model, stages I and II were difficult to identify, highlighting a limitation of the TNM staging system as a prognostic assessment tool.

To further investigate the role of nutritional immune status in CRC progression and to enhance the prognostic value of the PNI within the NIRS framework, we analyzed the correlation between the PNI and the maximum tumor diameter. Since NIRS integrates the PNI with tumor markers (CEA, CA19-9, and CA72-4), it provides an overall reflection of both nutritional immune status and tumor-related factors. However, in clinical datasets, patients may exhibit combinations such as low PNI with low tumor markers or high PNI with high tumor markers. In these cases, the resulting NIRS values may be similar, potentially limiting prognostic discrimination. Therefore, evaluating the independent prognostic significance of the PNI is essential.

Previous studies have reported inconsistent findings regarding the relationship between the PNI and the tumor diameter (25). While some studies have suggested that the PNI and the maximum tumor diameter are independent prognostic factors (9, 12, 25), our results indicate that maximum tumor diameter itself is not a risk factor in patients with CRC. Instead, we observed a negative correlation between the PNI and the maximum tumor diameter (Figure 5A). Further analysis showed that the maximum tumor diameter decreased more rapidly in survivors than in non-survivors, suggesting that survivors exhibit greater sensitivity of the nutritional immune response to tumor size. This may indicate that patients with good nutritional immune status maintain a rapid response to treatment and are more likely to achieve favorable survival outcomes. In situations where NIRS alone may not effectively discriminate prognostic differences among certain patients, the combined analysis of PNI and maximum tumor diameter can provide additional biological insights, facilitating a more comprehensive understanding of the interplay between nutritional immune status, tumor burden, and survival outcomes.

LOESS analysis further revealed distinctive patterns in the relationship between the PNI and the tumor size (Figure 5B). When the value of PNI was ≥50, the curve stabilized, indicating that patients with good nutritional immune status exhibited more stable tumor progression patterns. We speculate that adequate nutritional immune status may enhance immune surveillance and regulate tumor growth patterns (26). In contrast, when the value of PNI was < 50, the curve displayed greater fluctuations, suggesting increased heterogeneity in tumor progression among patients with poor nutritional immune status. Consistent with current understanding, patients with good nutritional immune status (PNI ≥ 50) who survived had smaller tumor diameters than non-survivors (27). However, under conditions of poor nutritional immune status (PNI < 50), survivors unexpectedly had larger tumors than non-survival patients. This observation highlights the complex interplay between host nutritional immune status and tumor behavior in CRC.

## 5 Conclusion

Based on the nutritional immune indicators and tumor markers (PNI, CEA, CA19-9, and CA72-4) combined with unsupervised learning methods, the novel NIRS model provides a new bioinformatic tool for the preoperative risk assessment and classification and postoperative prognostic assessment in patients with CRC. It is suitable for embedding in clinical pathways or decision support systems within the primary medical care system because of the high accessibility and cost-effectiveness of these indicators. Traditionally, PNI is negatively correlated with the maximum tumor diameter; however, our study indicates that the relationship may be more complex at low PNI levels than at high PNI levels. These findings may have significant implications for treatment strategies and postoperative management in patients with CRC.

Nevertheless, our study has certain limitations, and it is necessary to apply further NIRS models to other clinical databases to validate their generalizability. Furthermore, the complexity of the relationship between poor nutritional immune status and tumor diameter requires further investigation through both clinical and experimental studies. Overall, this study presents a novel NIRS model for patients with CRC from the perspective of nutritional immunity and reveals a previously underexplored relationship between the PNI and the maximum tumor diameter.

## Data Availability

The datasets presented in this study can be found in online repositories. The names of the repository/repositories and accession number(s) can be found at: https://zenodo.org/records/17217558/files/NutrImmune_data.xls?download=1.
