# Supplementary material for: A novel nutritional immune risk score model for long-term prognosis in colorectal cancer using clustering and principal component analysis
Source: Front Nutr. 2026 Apr 15;13:1734873. doi: 10.3389/fnut.2026.1734873 (PMC13124479; doi:10.3389/fnut.2026.1734873)
Supplement: Supplementary file 1 [file Supplementary_file_1.docx]

patients for analysis

(n = 892)

patients who received neoadjuvant radiotherapy or chemoradiotherapy before surgery (*n* = 153),

patients who had undergone other surgery prior to study enrollment (*n* = 34),

patients with a past or concurrent history of other malignancies (*n* = 19),

patients who discontinued treatment after diagnosis (*n* = 10),

patients who have experienced recurrence after surgery (*n* = 4),

patients with incomplete clinical data or who were lost to follow-up (*n* = 139).

patients aged over 18 years who underwent curative resection

(n = 1251)

Flowchart S1: The flowchart of study

| **TABLE S1. Results of K-S test and Chi-square test.** | | |
| --- | --- | --- |
| **Fold** | **KS test p-value** | **Chi-square test p-value** |
| 1 | 0.391 | 0.536 |
| 2 | 0.243 | 0.213 |
| 3 | <0.001 | 0.834 |
| 4 | 0.373 | 0.090 |
| 5 | 0.565 | 0.701 |
| 6 | 0.205 | 0.137 |
| 7 | 0.925 | 0.825 |
| 8 | 0.675 | 0.421 |

| **TABLE S2. Nutritional immune risk score (NIRS) coefficient.** | | | | | |
| --- | --- | --- | --- | --- | --- |
| **Variable** | **PC1** | **PC2** | **Weights** | | **Coefficient** |
| PNI | 0.032 | 0.996 | PC1:0.44 | PC2:0.56 | 0.572 |
| CEA | -0.136 | -0.073 | PC1:0.44 | PC2:0.56 | -0.101 |
| CA19-9 | -0.990 | 0.043 | PC1:0.44 | PC2:0.56 | -0.412 |
| CA72-4 | -0.036 | -0.021 | PC1:0.44 | PC2:0.56 | -0.028 |

| TABLE S3. Maximum Tumor Diameter (cm) by Survival Status Across TNM Stages and PNI Groups. | | | | | | | |
| --- | --- | --- | --- | --- | --- | --- | --- |
|  |  | **PNI < 50** | |  | **PNI ≥ 50** | |  |
|  |  | **survival (n)** | **non-survival(n)** | ***p*** | **survival (n)** | **non-survival (n)** | ***p*** |
| TNM | I | 4.81 (34) | 4.33 (3) | 0.737 | 3.50 (108) | 3.77 (11) | 0.48888 |
|  | II | 5.74 (124) | 5.42 (37) | 0.758 | 4.60 (148) | 3.80 (20) | 0.028 |
|  | III | 5.79 (67) | 4.70 (64) | 0.001 | 4.12 (136) | 4.62 (82) | 0.059 |
|  | IV | 5.50 (5) | 5.57 (21) | 0.819 | 2.65 (2) | 4.58 (30) | 0.036 |
